# Supplementary figures and images for: Temperature alters the shape of predator–prey cycles through effects on underlying mechanisms
Source: PeerJ. 2020 Jun 19;8:e9377. doi: 10.7717/peerj.9377 (PMC7307560; doi:10.7717/peerj.9377)

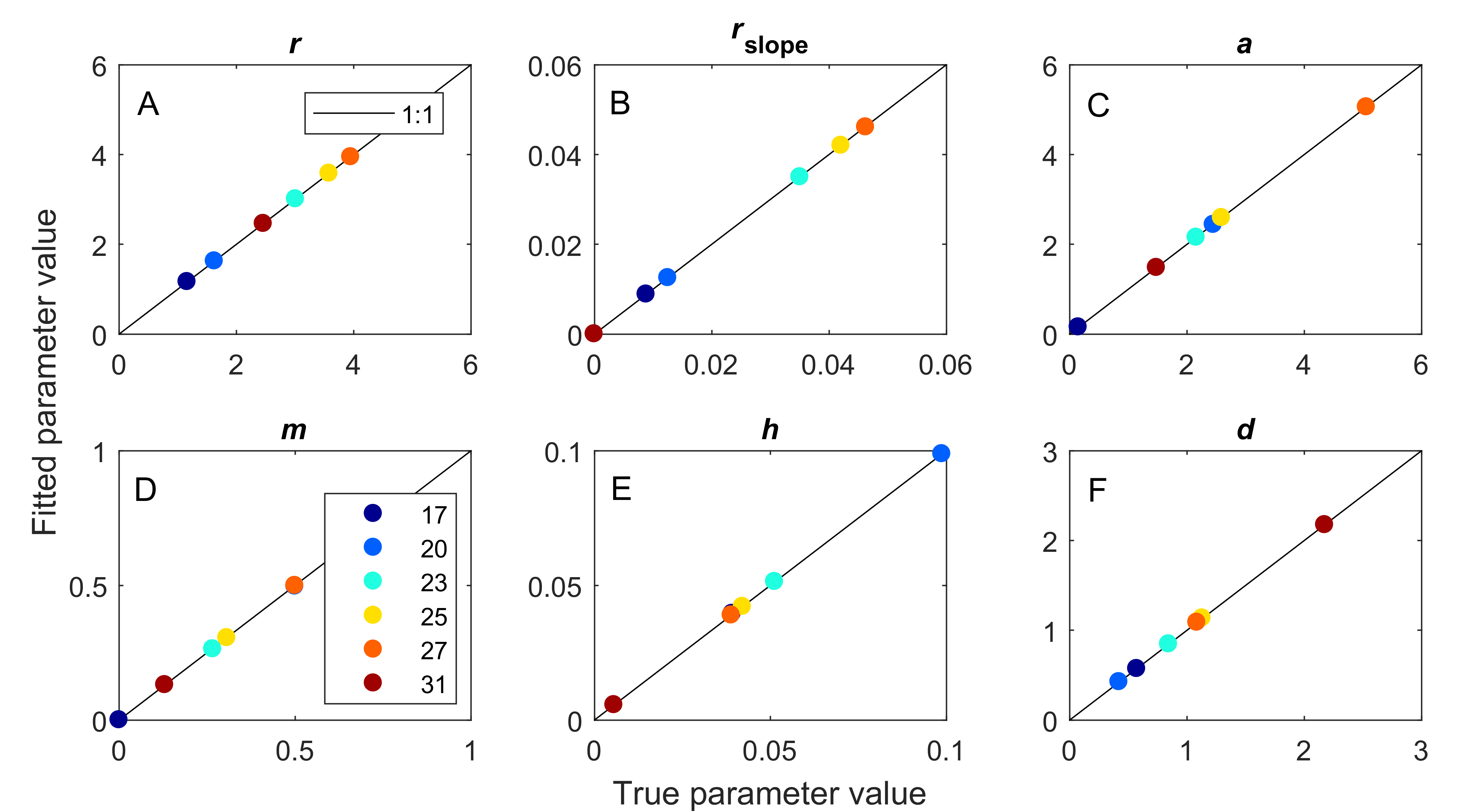

Supplement: Supplemental Information 5 — Fitting our model to simulated dynamics returned parameter values that were a good match to true values used in generating simulations. This was true across six temperatures for all six fitted parameters, (A) maximum prey population growth rate, (B) the strength of density dependance in prey population growth, (C) space clearance rate, (D) interference among predators, (E) handling time, and (F) predator mortality rate. [file peerj-08-9377-s005.png]

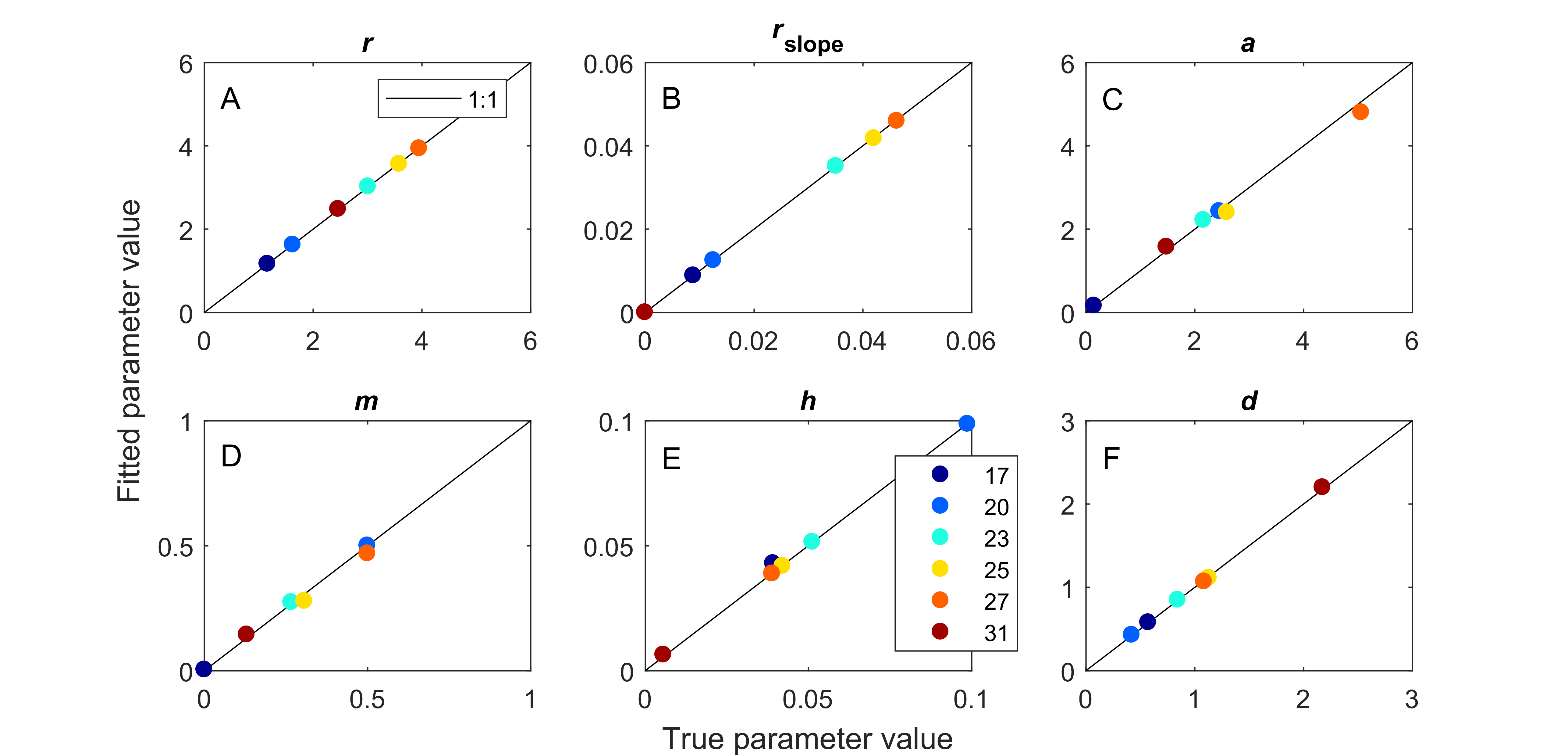

Supplement: Supplemental Information 6 — Fitting our model to simulated dynamics with noise returned parameter values that were a good match to true values used in generating simulations. Fitted parameters are average of 10 replicated simulations. This was true across six temperatures for all six fitted parameters, (A) maximum prey population growth rate, (B) the strength of density dependance in prey population growth, (C) space clearance rate, (D) interference among predators, (E) handling time, and (F) predator mortality rate. [file peerj-08-9377-s006.png]
